# Serum 25-hydroxyvitamin D_3_ Levels and Diabetes in a Japanese Population: The DOSANCO Health Study

**DOI:** 10.2188/jea.JE20210007

**Published:** 2023-01-05

**Authors:** Koshi Nakamura, Shu-Ping Hui, Shigekazu Ukawa, Emiko Okada, Takafumi Nakagawa, Akihiro Imae, Hiroaki Okabe, Zhen Chen, Yusuke Miura, Hitoshi Chiba, Akiko Tamakoshi

**Affiliations:** 1 Department of Public Health and Hygiene, Graduate School of Medicine, University of the Ryukyus, Okinawa, Japan; 2 Department of Public Health, Hokkaido University Faculty of Medicine, Sapporo, Japan; 3 Faculty of Health Sciences, Hokkaido University, Sapporo, Japan; 4 Research Unit of Advanced Interdisciplinary Care Science, Osaka City University Graduate School of Human Life Science, Osaka, Japan; 5 Department of Nutritional Epidemiology and Shokuiku, National Institute of Biomedical Innovation, Health and Nutrition, Tokyo, Japan; 6 The Hokkaido Centre for Family Medicine, Sapporo, Japan; 7 Suttu Municipal Clinic, Hokkaido, Japan; 8 School of Medical Technology, Faculty of Health Science, Gunma Paz University, Takasaki, Japan; 9 Department of Nutrition, Sapporo University of Health Sciences, Sapporo, Japan

**Keywords:** 25-hydroxyvitamin D_3_, diabetes, insulin resistance, Japanese

## Abstract

**Background:**

Both decreased insulin sensitivity and impaired insulin secretion are common in Asian populations with diabetes, in contrast to Western populations. There is limited evidence regarding the association between insulin response in diabetes in Asian populations and serum 25-hydroxyvitamin D_3_ (25[OH]D_3_) insufficiency.

**Methods:**

The present cross-sectional study compared the prevalence of diabetes, defined as a fasting plasma glucose level ≥126 mg/dL and/or a HbA1c level ≥6.5%, among 480 participants aged 35–79 years not taking anti-diabetes medications, based on serum 25(OH)D_3_ levels. A logistic regression model was used to calculate the odds ratios for diabetes in each serum 25(OH)D_3_ group. Furthermore, this study examined the association between serum 25(OH)D_3_ levels and the index of homeostasis model assessment of insulin resistance (HOMA-IR) using a linear regression model.

**Results:**

The prevalence of diabetes was 7.29% in the study population, and was higher in lower serum 25(OH)D_3_ quartile groups. The odds ratios for diabetes in the first, second, and third serum 25(OH)D_3_ quartile groups (25[OH]D_3_: ≤18.10, 18.11–22.90, and 22.91–28.17 ng/mL) were 4.02 (95% confidence interval [CI], 1.25–12.92), 2.50 (95% CI, 0.77–8.10), and 1.91 (95% CI, 0.60–6.09), respectively, with the fourth quartile group ( ⩾ 28.18 ng/mL) serving as the reference group, after adjusting for sociodemographic, lifestyle, physical and environmental factors. Serum 25(OH)D_3_ levels showed an inverse association with log-transformed HOMA-IR after adjusting for similar factors (standardized β = −0.08; 95% CI, −0.14 to −0.02).

**Conclusion:**

Serum 25(OH)D_3_ levels were inversely associated with diabetes prevalence in a general Japanese population, with a slight inverse association between serum 25(OH)D_3_ levels and HOMA-IR.

## INTRODUCTION

There has been increasing interest in elucidating the biological effects of vitamin D beyond the effects on the musculoskeletal system. Lack of vitamin D is thought to be involved in the pathogenesis of a number of disorders via inactivation of vitamin D receptors located in various organs.^1^^–^^3^ This is also true for diabetes, because vitamin D receptors are present in pancreatic β-cells, responsible for insulin secretion, as well as peripheral target tissues (ie, skeletal muscles and adipose tissues), responsible for glucose homeostasis by responding to insulin.^2^^–^^4^ 25-hydroxyvitamin D (25[OH]D), of which the majority is 25(OH)D_3_,^5^^,^^6^ is the circulating form of vitamin D and, when needed, is converted to the biologically active form to exert many physiological functions.^2^^,^^3^

A recent epidemiological study suggests that low levels of serum 25(OH)D_3_, but not 25(OH)D_2_, are associated with incident diabetes in Western populations,^5^ demonstrating the need to focus on serum 25(OH)D_3_ levels when evaluating vitamin D in the body with regard to diabetes pathogenesis. A few Asian studies also suggest the existence of an inverse association between serum 25(OH)D_3_ levels and the prevalence or incidence of diabetes.^6^^,^^7^ However, studies to better understand the association between serum 25(OH)D_3_ insufficiency and insulin response in diabetes in Asian populations have been limited.^8^^–^^10^ Both decreased insulin sensitivity (ie, insulin resistance) and impaired insulin secretion are common insulin responses in diabetes in Asian populations,^11^^,^^12^ unlike in Western populations, in whom diabetes is largely attributable to insulin resistance.^13^^,^^14^ Therefore, we conducted a cross-sectional study to investigate the association between serum 25(OH)D_3_ levels and diabetes in a general Japanese population.

## METHODS

### Study design and population

A cross-sectional study was conducted as part of the Dynamics of Lifestyle and Neighborhood Community on Health Study (DOSANCO Health Study), a community-based study involving 2,100 participants aged ≥3 years in the town of Suttu, Hokkaido, Japan (response, 79.6% of all residents at home) during 2015.^15^ Suttu gets approximately 1,400 hours of sunlight throughout the year, which is less than the average level in Japan (approximately 1,900 hours).^16^ It usually snows between late November and early April in Suttu.^16^ Herein, we undertook a detailed survey of Suttu residents aged 35–79 years to obtain evidence that may contribute to the prevention of metabolic disorders and atherosclerotic disease. Of 1,686 residents aged 35–79 years, 1,379 (650 men and 729 women) completed a self-administered questionnaire (response, 81.8%), and 545 (245 men and 300 women) underwent additional physical measurements and provided blood samples between the months of August and November (response, 32.3%). Of the 545 participants, 65 were deemed ineligible for inclusion for the following reasons: taking medications to control their diabetes (*n* = 53), possible inappropriate data on total daily energy intake of <500 kcal/day or >5,000 kcal/day (*n* = 2), missing data related to diabetes (ie, glucose, insulin, and medical history) (*n* = 5), or missing data related to characteristics other than vitamin D and diabetes (*n* = 5). We excluded those individuals with treated diabetes, because they may have modified their lifestyles with regard to vitamin D levels (eg, sunlight exposure through outdoor exercise, dietary intake), which may have led to an underestimation of the association of interest. The remaining 480 individuals that were not taking anti-diabetes medications (220 men and 260 women) were considered eligible study participants and included in the subsequent analyses. The study protocol was approved by the Institutional Review Committee for Ethical Issues of the Faculty of Medicine (15-002, 16-007) and the Faculty of Health Sciences (16-10), Hokkaido University. Written informed consent was obtained from all participants. The present analysis was also approved by the Institutional Review Committee for Ethical Issues of University of the Ryukyus (1643, 1644).

### Data collection

Venous blood samples were collected by cubital venipuncture after an overnight fast. Some blood samples were then transported to a commercial laboratory (Daiichi Kishimoto Clinical Laboratories, Inc., Sapporo, Japan) to measure fasting plasma glucose, glycated hemoglobin (HbA1c), and serum creatinine levels using standardized methods. Serum of the remaining blood samples was separated, centrifuged after blood coagulation, and then stored at −80°C until vitamin D and insulin levels were measured. Serum 25(OH)D_3_ (ng/mL) measurements were performed at our laboratory (Hokkaido University Faculty of Health Science) using liquid chromatography with tandem mass spectrometry (LC-MS/MS).^17^ Fasting serum insulin levels (µU/mL) were measured via a chemiluminescent immunoassay using an automatic analyzer (ARCHITECT i2000; Abbott Japan Co., Ltd., Tokyo, Japan) at another commercial laboratory (LSI Medience Corporation, Tokyo, Japan). Insulin resistance was estimated via the homeostasis model assessment of insulin resistance (HOMA-IR) using the following formula: {fasting glucose (mg/dL) × fasting insulin (µU/mL)}/405.^18^ Impaired insulin secretion was estimated via the homeostasis model assessment of β-cell function (HOMA-β) using the following formula: {360 × fasting insulin (µU/mL)}/{fasting glucose (mg/dL) − 63}.^18^

Body height and weight were measured, and body mass index was calculated as weight (kg)/height squared (m^2^). Estimated glomerular filtration rate (mL/min/1.73 m^2^) was calculated using serum creatinine data with the Chronic Kidney Disease Epidemiology Collaboration equation,^19^ modified by the Japanese coefficient.^20^ Other data collected using the self-administered questionnaire included age, sex, work status, exercise and smoking habits, and dietary intake. Habitual exercise was classified as partaking in ≥10 min of physical exercise at least once a week.^21^ Smoking habits were classified according to whether a participant had never smoked, was a former smoker, or was a current smoker. Dietary habits during the past month were assessed using a validated brief self-administered diet history questionnaire (BDHQ).^22^^,^^23^ Briefly, the BDHQ is a 10-page fixed-portion questionnaire used to estimate dietary intake of 58 food items. The food items and portion sizes comprising the BDHQ were derived primarily from a food list used in the National Health and Nutrition Survey in Japan and from several Japanese recipe books.

### Statistical analysis

Initially, we compared the prevalence of diabetes, defined as a fasting plasma glucose level ≥126 mg/dL and/or a HbA1c level ≥6.5%,^24^ in study participants that were not taking anti-diabetes medications, grouped according to quartiles of serum 25(OH)D_3_ levels. Because the clinical threshold of serum 25(OH)D_3_ has not been established, we used quartiles to categorize serum 25(OH)D_3_ levels for the comparison of interest. Then, odds ratios with corresponding 95% confidence intervals (CIs) were calculated for the presence of diabetes using a logistic regression model for each study group categorized by quartile of serum 25(OH)D_3_ levels. The group with the highest quartile of serum 25(OH)D_3_ levels was taken as the reference group. The model incorporated the following covariates as potential confounding factors: age (years, as a continuous variable), sex (male or female), months during which blood samples were collected (August–September or October–November) (model 1), smoking habits (current, former, or never smoker, using two dummy variables with never smoker as the reference), estimated glomerular filtration rate (mL/min/1.73 m^2^, as a continuous variable), work status (yes or no), exercise habits (yes or no), alcohol intake (g/day, as a continuous variable), protein intake (g/day, as a continuous variable), fat intake (g/day, as a continuous variable), carbohydrate intake (g/day, as a continuous variable), total dietary fiber intake (g/day, as a continuous variable) (model 2), and body mass index (kg/m^2^, as a continuous variable) (model 3). In addition, we compared the prevalence of diabetes in study participants grouped according to sex-specific quartiles of serum 25(OH)D_3_ levels to eliminate the confounding effect of sex due to different distributions of serum 25(OH)D_3_ levels between sexes. The prevalence of diabetes was also calculated for participants grouped according to the common criteria of vitamin D insufficiency (ie, <19.9, 20–29.9, and ≥30 ng/mL).^2^ A similar logistic regression model was employed to calculate odds ratios for each serum 25(OH)D_3_ group. Furthermore, we tested the trend for a linear association between serum 25(OH)D_3_ levels and diabetes, and calculated odds ratio with corresponding 95% CIs for the presence of diabetes associated with a one standard deviation decrease in serum 25(OH)D_3_ levels, using a similar logistic regression model that incorporated the same covariates in addition to a continuous variable of serum 25(OH)D_3_ (ng/mL) instead of the categorical variables of serum 25(OH)D_3_ quartiles.

Next, to explore the association between serum 25(OH)D_3_ insufficiency and insulin response in diabetes, we compared HOMA-IR and HOMA-β in study participants grouped according to quartiles of serum 25(OH)D_3_ levels. The Kruskal-Wallis test was used to compare each index crudely. Subsequently, analysis of covariance, which incorporated the same covariates used in the earlier logistic regression model evaluating the presence of diabetes, was used to compare both log-transformed HOMA-IR and log-transformed HOMA-β, because of skewed distributions of the HOMA-IR and HOMA-β data. Adjusted geometric means of each index were presented for each serum 25(OH)D_3_ quartile group. Moreover, a linear regression model was also employed to examine the association between serum 25(OH)D_3_ levels and each log-transformed index in the study participants, incorporating the same covariates. To explore the association between serum 25(OH)D_3_ insufficiency and insulin response in pre-diabetes, similar analyses were repeated after excluding those study participants with diabetes, per the above definition.^24^ Similar analyses were repeated among the normo-to-prediabetic participants stratified by the absence or presence of obesity, defined as a body mass index ≥25 kg/m^2^,^25^ as the insulin response in abnormal glucose metabolism depends partially upon obesity status.^11^^,^^12^

Analyses were performed using Stata 15 (StataCorp LP, College Station, TX, USA). All probability values were two-tailed, and the significance level was set at *P* < 0.05.

## RESULTS

### Characteristics of the study population

The mean age of the 480 study participants was 57.9 (standard deviation [SD], 12.5) years. The mean serum 25(OH)D_3_ concentration was 23.5 (SD, 8.12) ng/mL for the overall population. Some characteristics were significantly different among groups of participants categorized by serum 25(OH)D_3_ ranges according to quartile concentrations (Table 1). Age and alcohol intake tended to be lower in lower serum 25(OH)D_3_ groups, whereas the estimated glomerular filtration rates tended to be higher in lower serum 25(OH)D_3_ groups. The percentages of females and participants with vitamin D measurements in autumn months tended to be higher in lower serum 25(OH)D_3_ groups. Total dietary energy, protein, and dietary vitamin D intake were lowest in the lowest serum 25(OH)D_3_ group.

**Table 1.** Characteristics of study participants

	Overall (*N* = 480)	Serum 25-hydroxyvitamin D_3_, ng/mL				*P*-value for difference

		1st quartile group	2nd quartile group	3rd quartile group	4th quartile group	
		≤18.10 (2.08–18.10)	18.11–22.90	22.91–28.17	≥28.18 (28.18–78.83)	
		(*n* = 120)	(*n* = 120)	(*n* = 120)	(*n* = 120)	
Age, years	57.9 (12.5)	55.3 (12.2)	56.2 (13.2)	58.8 (12.1)	61.2 (11.6)	0.001
Women	54.2% (260)	61.7% (74)	58.3% (70)	53.3% (64)	43.3% (52)	0.03
Months of blood sample collection						<0.001
August–September	34.0% (163)	28.3% (34)	24.2% (29)	31.7% (38)	51.7% (62)	
October–November	66.0% (317)	71.7% (86)	75.8% (91)	68.3% (82)	48.3% (58)	
Smoking habits						0.10
Never smoker	45.8% (220)	49.2% (59)	53.3% (64)	45.8% (55)	35.0% (42)	
Former smoker	31.9% (153)	27.5% (33)	25.8% (31)	32.5% (39)	41.7% (50)	
Current smoker	22.3% (107)	23.3% (28)	20.8% (25)	21.7% (26)	23.3% (28)	
Estimated glomerular filtration rate, mL/min/1.73 m^2^	81.0 (11.2)	82.7 (10.5)	82.1 (11.5)	80.3 (11.4)	78.8 (11.2)	0.03
Working	68.8% (330)	72.5% (87)	70.8% (85)	68.3% (82)	63.3% (76)	0.44
Regular exercise	41.3% (198)	31.7% (38)	43.3% (52)	44.2% (53)	45.8% (55)	0.10
Total dietary energy intake, kcal/day	1,787 (578)	1,706 (598)	1,790 (587)	1,845 (554)	1,807 (568)	0.30
Alcohol intake, g/day	14.6 (25.3)	9.2 (19.7)	13.8 (26.3)	16.6 (27.8)	18.9 (25.7)	0.02
Protein intake, g/day	67.1 (28.5)	60.9 (27.6)	67.4 (29.8)	71.3 (30.1)	69.0 (25.5)	0.03
Fat intake, g/day	50.0 (19.9)	48.6 (21.0)	50.6 (20.4)	52.2 (20.2)	48.6 (18.0)	0.44
Carbohydrate intake, g/day	235.4 (82.1)	234.4 (84.9)	236.2 (81.0)	237.3 (76.6)	233.5 (86.7)	0.99
Total dietary fiber intake, g/day	11.0 (5.4)	10.4 (5.8)	11.3 (5.5)	11.4 (5.2)	11.1 (5.1)	0.48
Dietary vitamin D intake, µg/day	15.7 (12.9)	12.2 (9.9)	15.5 (13.0)	18.4 (15.4)	16.6 (12.1)	0.002
Body mass index, kg/m^2^	23.7 (3.6)	23.9 (4.1)	23.8 (3.4)	23.5 (3.6)	23.4 (3.4)	0.61

Data are mean (standard deviation), or % (number) of participants.

One-way analysis of variance, or Chi-square test was used to compare each characteristic in each serum 25-hydroxyvitamin D_3_ quartile group.

### Serum 25(OH)D_3_ levels and diabetes prevalence

Participants classified as having diabetes accounted for 7.29% (*n* = 35) of the study population with median HOMA-IR and HOMA-β of 0.99 and 52.2, respectively (data not shown). The prevalence of diabetes tended to be higher in lower serum 25(OH)D_3_ groups (Table 2). The odds ratios for diabetes were 4.02 (95% CI, 1.25–12.92) for the first, 2.50 (95% CI, 0.77–8.10) for the second, and 1.91 (95% CI, 0.60–6.09) for the third serum 25(OH)D_3_ quartile group, compared with the fourth quartile group (as the reference), after adjusting for major potential confounding factors including body mass index (model 3). There was an inverse linear association between serum 25(OH)D_3_ levels and the prevalence of diabetes (*P* = 0.047); the odds ratio for diabetes associated with every one SD (8.12 ng/mL) decrease in serum 25(OH)D_3_ level was 1.57 (95% CI, 1.00–2.43), after adjusting for major potential confounding factors including body mass index (data not shown).

**Table 2.** Odds ratios for diabetes in participants grouped by serum 25-hydroxyvitamin D_3_ quartiles

	Serum 25-hydroxyvitamin D_3_, ng/mL			

	1st quartile group	2nd quartile group	3rd quartile group	4th quartile group
	≤18.10 (2.08–18.10)	18.11–22.90	22.91–28.17	≥28.18 (28.18–78.83)
	(*n* = 120)	(*n* = 120)	(*n* = 120)	(*n* = 120)
Cases of diabetes, *n*	12	9	8	6
Prevalence, %	10.0	7.5	6.7	5.0
Adjusted odds ratio (95% CI), model 1	3.14 (1.08–9.11)	2.06 (0.68–6.24)	1.63 (0.53–4.98)	1.00 (Reference)
Adjusted odds ratio (95% CI), model 2	4.02 (1.25–12.89)	2.48 (0.76–8.05)	1.89 (0.59–6.02)	1.00 (Reference)
Adjusted odds ratio (95% CI), model 3	4.02 (1.25–12.92)	2.50 (0.77–8.10)	1.91 (0.60–6.09)	1.00 (Reference)

CI, confidence interval.

Diabetes was defined as a fasting plasma glucose level ≥126 mg/dL and/or a HbA1c level ≥6.5% [reference 24].

Three different logistic regression models were used to calculate odds ratio (95% CI) with the 4th quartile group serving as the reference group: model 1 adjusted for age, sex, and months of blood sample collection; model 2 adjusted for the same covariates used in model 1, in addition to smoking habits, estimated glomerular filtration rate, work status, exercise habits, alcohol intake, protein intake, fat intake, and total dietary fiber intake; model 3 adjusted for the same covariates used in model 2, in addition to body mass index.

A similar pattern was observed for odds ratios in study participants grouped according to sex-specific quartiles of serum 25(OH)D_3_ levels (eTable 1), as well as in study participants grouped according to the common criteria of vitamin D insufficiency (eTable 2).

### Serum 25(OH)D_3_ levels, HOMA-IR, and HOMA-β

The median of HOMA-IR tended to be slightly higher in lower serum 25(OH)D_3_ groups, with no significant difference among the four serum 25(OH)D_3_ groups (Table 3). The lowest serum 25(OH)D_3_ quartile group exhibited the highest geometric mean of HOMA-IR among the four groups (after adjusting for major potential confounding factors other than body mass index), whereas the highest serum 25(OH)D_3_ quartile group exhibited the lowest geometric mean of HOMA-IR (model 2). However, further adjusting for body mass index in the analysis did not show a pattern similar to that observed in model 2 (model 3).

**Table 3.** Median and geometric means of HOMA-IR and HOMA-β in participants grouped by serum 25-hydroxyvitamin D_3_ quartiles

	Serum 25-hydroxyvitamin D_3_, ng/mL				*P*-value

	1st quartile group	2nd quartile group	3rd quartile group	4th quartile group	
	≤18.10 (2.08–18.10)	18.11–22.90	22.91–28.17	≥28.18 (28.18–78.83)	
	(*n* = 120)	(*n* = 120)	(*n* = 120)	(*n* = 120)	
HOMA-IR					
Median (interquartile range)	1.06 (0.72–1.74)	0.98 (0.63–1.42)	0.98 (0.66–1.74)	0.92 (0.55–1.53)	0.43
Adjusted geometric mean (95% CI), model 1	1.21 (1.01–1.46)	1.07 (0.89–1.28)	1.14 (0.95–1.36)	0.98 (0.84–1.16)	0.15
Adjusted geometric mean (95% CI), model 2	1.20 (0.93–1.53)	1.06 (0.83–1.36)	1.14 (0.89–1.46)	0.99 (0.77–1.25)	0.20
Adjusted geometric mean (95% CI), model 3	1.07 (0.87–1.32)	0.98 (0.79–1.20)	1.09 (0.88–1.34)	0.95 (0.77–1.16)	0.21
HOMA-β					
Median (interquartile range)	52.5 (37.4–82.5)	52.0 (36.0–75.7)	53.5 (34.7–80.8)	51.6 (30.3–73.0)	0.66
Adjusted geometric mean (95% CI), model 1	52.1 (44.4–61.1)	51.3 (43.7–60.3)	53.2 (45.6–62.2)	48.8 (42.4–56.1)	0.75
Adjusted geometric mean (95% CI), model 2	52.9 (42.7–65.4)	52.6 (42.5–65.1)	55.1 (44.4–68.2)	51.0 (41.4–62.9)	0.83
Adjusted geometric mean (95% CI), model 3	48.7 (40.3–58.9)	49.3 (40.8–59.6)	53.3 (44.1–64.4)	49.5 (41.2–59.6)	0.58

CI, confidence interval.

The Kruskal-Wallis test, or analysis of covariance with three different models was used to compare HOMA-IR and HOMA-β in each serum 25-hydroxyvitamin D_3_ quartile group: model 1 adjusted for age, sex, and months of blood sample collection; model 2 adjusted for the same covariates used in model 1, in addition to smoking habits, estimated glomerular filtration rate, work status, exercise habits, alcohol intake, protein intake, fat intake, and total dietary fiber intake; model 3 adjusted for the same covariates used in model 2, in addition to body mass index.

Overall, there was a slight inverse association between serum 25(OH)D_3_ levels and log-transformed HOMA-IR, even after adjusting for major potential confounding factors, including body mass index (standardized β = −0.08; 95% CI, −0.14 to −0.02) (Table 4, model 3). However, there was no apparent association between serum 25(OH)D_3_ levels and log-transformed HOMA-β, after adjusting for major potential confounding factors.

**Table 4.** Standardized β coefficients between serum 25-hydroxyvitamin D_3_ and ln(HOMA-IR) and ln(HOMA-β) in the study population

	Ln(HOMA-IR)		Ln(HOMA-β)	
Overall (*N* = 480)				
Serum 25-hydroxyvitamin D_3_, ng/mL				
Adjusted standardized β coefficient (95% CI), model 1	−0.11 (−0.17 to −0.04)	*P* = 0.001	−0.06 (−0.11 to 0.001)	*P* = 0.06
Adjusted standardized β coefficient (95% CI), model 2	−0.11 (−0.17 to −0.04)	*P* = 0.002	−0.05 (−0.11 to 0.01)	*P* = 0.12
Adjusted standardized β coefficient (95% CI), model 3	−0.08 (−0.14 to −0.02)	*P* = 0.006	−0.03 (−0.08 to 0.03)	*P* = 0.31
Normo-to-prediabetic (*N* = 445)				
Serum 25-hydroxyvitamin D_3_, ng/mL				
Adjusted standardized β coefficient (95% CI), model 1	−0.11 (−0.17 to −0.04)	*P* = 0.001	−0.07 (−0.12 to −0.01)	*P* = 0.02
Adjusted standardized β coefficient (95% CI), model 2	−0.10 (−0.17 to −0.03)	*P* = 0.003	−0.06 (−0.12 to 0.0002)	*P* = 0.05
Adjusted standardized β coefficient (95% CI), model 3	−0.08 (−0.14 to −0.02)	*P* = 0.005	−0.04 (−0.09 to 0.01)	*P* = 0.11
Non-obese, normo-to-prediabetic (*N* = 303)				
Serum 25-hydroxyvitamin D_3_, ng/mL				
Adjusted standardized β coefficient (95% CI), model 1	−0.08 (−0.15 to −0.02)	*P* = 0.02	−0.05 (−0.11 to 0.01)	*P* = 0.09
Adjusted standardized β coefficient (95% CI), model 2	−0.08 (−0.15 to −0.01)	*P* = 0.03	−0.04 (−0.10 to 0.02)	*P* = 0.22
Adjusted standardized β coefficient (95% CI), model 3	−0.07 (−0.14 to −0.01)	*P* = 0.03	−0.04 (−0.10 to 0.02)	*P* = 0.24
Obese, normo-to-prediabetic (*N* = 142)				
Serum 25-hydroxyvitamin D_3_, ng/mL				
Adjusted standardized β coefficient (95% CI), model 1	−0.09 (−0.20 to 0.02)	*P* = 0.10	−0.05 (−0.15 to 0.05)	*P* = 0.32
Adjusted standardized β coefficient (95% CI), model 2	−0.10 (−0.22 to 0.01)	*P* = 0.08	−0.05 (−0.16 to 0.05)	*P* = 0.29
Adjusted standardized β coefficient (95% CI), model 3	−0.09 (−0.19 to 0.02)	*P* = 0.11	−0.04 (−0.14 to 0.05)	*P* = 0.39

CI, confidence interval; HOMA-IR, homeostasis model assessment of insulin resistance; HOMA-β, homeostasis model assessment of β-cell function.

Normo-to-prediabetes was defined as a fasting plasma glucose level <126 mg/dL and a HbA1c level <6.5% [reference 24].

Obesity was defined as a body mass index ≥25 kg/m^2^ [reference 25].

Three different linear regression models were used to examine the standardized β coefficient: model 1 adjusted for age, sex, and months of blood sample collection; model 2 adjusted for the same covariates used in model 1, in addition to smoking habits, estimated glomerular filtration rate, work status, exercise habits, alcohol intake, protein intake, fat intake, and total dietary fiber intake; model 3 adjusted for the same covariates used in model 2, in addition to body mass index.

When we restricted the study population to the 445 participants who were normo-to-prediabetic (ie, a fasting plasma glucose level <126 mg/dL and a HbA1c level <6.5%), there was also a slight inverse association between serum 25(OH)D_3_ levels and log-transformed HOMA-IR (standardized β = −0.08; 95% CI, −0.14 to −0.02). Similar slight inverse associations were observed between serum 25(OH)D_3_ levels and log-transformed HOMA-IR (standardized β = −0.07; 95% CI, −0.14 to −0.01) among the 303 non-obese, normo-to-prediabetic participants, as well as between serum 25(OH)D_3_ levels and log-transformed HOMA-IR (standardized β = −0.09; 95% CI, −0.19 to 0.02) among the 142 obese, normo-to-prediabetic participants.

The magnitude of adjusted standardized β coefficient between log-transformed HOMA-IR and serum 25(OH)D_3_ levels was less than one-third of that between body mass index and serum 25(OH)D_3_ levels (eTable 3, calculated using the multivariate linear regression model).

## DISCUSSION

Study participants with lower serum 25(OH)D_3_ levels had a significantly higher likelihood for the presence of diabetes in a community-based Japanese adult population who were not taking anti-diabetes medications, after adjustment for major potential confounding factors, such as sociodemographic, lifestyle, physical, and environmental factors. The presence of diabetes increased linearly with decreasing serum 25(OH)D_3_ levels, even within serum 25(OH)D_3_ levels considered clinically normal. Along with the link between low serum 25(OH)D_3_ levels and diabetes, serum 25(OH)D3 levels had a weaker as compared with body mass index, but significant, inverse association with HOMA-IR.

Chailurkit et al^6^ conducted a cross-sectional study of 2,641 community-based Thai individuals (mean age, 40.3 years), and reported that mean serum 25(OH)D_3_ levels were lower in those individuals with diabetes than in those without diabetes for older individuals in an urban area, but not for other demographics. Akter et al^7^ conducted a nested case-control study in working Japanese individuals (336 diabetic cases and 668 controls; mean age, 51 years), and reported that the odds ratios for incident diabetes were 0.68 (95% CI, 0.46–1.02) for the serum 25(OH)D_3_ 15.5–19.5 ng/mL group, 0.67 (95% CI, 0.44–1.03) for the serum 25(OH)D_3_ 19.6–24.2 ng/mL group, and 0.58 (95% CI, 0.36–0.92) for the serum 25(OH)D_3_ >24.2 ng/mL group, compared with the serum 25(OH)D_3_ <15.5 ng/mL group, after adjusting for major potential confounding factors other than body mass index (*P* for trend = 0.03). However, the association was no longer statistically significant after further adjusting for body mass index.^7^ The results of our study are somewhat consistent with the results of these previous Asian studies. On the other hand, Bi et al^10^ and Hidayat et al^26^ reported null associations of interest among individuals in Singapore (*N* = 114) and Indonesia (*N* = 78), respectively.

The previous Singapore cross-sectional study observed a slight inverse correlation between serum 25(OH)D_3_ levels and HOMA-IR in Asians, without allowing for adjustments for potential confounding factors (*N* = 114, *r* = −0.27, *P* = 0.003).^10^ The two previous Chinese cross-sectional studies, on the other hand, did not find any significant associations of interest in Asians, allowing only for adjustments for age and body mass index (*N* = 567, *r* = −0.025, *P* = 0.558)^8^ (*N* = 451, *r* = −0.068, *P* = 0.148).^9^ Results of our study showed a slight, but significant, inverse association of interest in an Asian population, even after adjusting for several potential confounding factors, although the magnitude of association was small in contrast to the moderately positive association between body mass index and HOMA-IR. On the other hand, we observed a null association between serum 25(OH)D_3_ levels and HOMA-β in Asians, even in non-obese individuals in whom early impairment of insulin secretion also commonly occurs as a result of low insulin secretion capacity.^11^^,^^12^ However, the HOMA-β results of our study should be interpreted with caution. HOMA-β is an alternative index of insulin secretion from pancreatic β-cells, with measurements of glucose and insulin obtained only during the fasting state,^18^ so it is less sensitive for detection of impaired insulin secretion compared with the gold standard indices (eg, insulinogenic index and insulin secretion sensitivity index-2), in which glucose and insulin are measured during both fasting and glucose-stimulated states.^27^^,^^28^ Furthermore, HOMA-IR and HOMA-β compete as a result of the shared use of fasting insulin. These issues may partially explain the somewhat weak link observed between vitamin D_3_ insufficiency and insulin resistance, despite the moderate link between vitamin D_3_ insufficiency and diabetes. Nevertheless, to the best of our knowledge, our study is the first to show the detailed nature of impaired glucose homeostasis associated with vitamin D_3_ insufficiency in Asians.

Physical activity, which is usually performed outdoors, prevents diabetes independent of body weight.^29^ Adequate exposure to sunlight during outdoor activities can contribute to the synthesis of vitamin D_3_ in the body.^30^ Moreover, results of our study suggest that high levels of vitamin D_3_ lead to prevention of diabetes, independent of exercise. When interpreting the results of our study together with the results of these previous studies, it is plausible that outdoor activity may have an additional benefit (compared with indoor activity) on diabetes prevention beyond physical activity itself, due to the biological effects of vitamin D_3_ synthesized in response to sunlight. Therefore, outdoor activity with sunlight exposure should be recommended over indoor activity for the prevention of diabetes, where possible.

The present study had several strengths, including use of a marker of vitamin D_3_, not total vitamin D as used in earlier studies, the size of the study population, which was larger than or similar to previous studies examining the association between serum 25(OH)D_3_ levels and HOMA-IR, and the consideration of potential confounding factors, especially dietary intake. However, the present study also had several limitations. First, the cross-sectional design could not guarantee prospective confirmation that serum 25(OH)D_3_ insufficiency causes diabetes. Second, the classification of the type of diabetes (ie, type 1 and type 2 diabetes) was not taken into account due to lack of available data. Study participants ranged from middle-aged to pre-senile, and diabetes was identified based on a single blood examination. Therefore, the majority of diabetes cases in this study were likely to be type 2 diabetes. Third, our study population comprised residents in a single northern rural community of Japan, which gets relatively short hours of sunlight throughout the year and snow during winter.^16^ Lifestyle and environmental factors in this community may determine vitamin D levels in the study population, and such factors can also influence glucose metabolism. Therefore, caution is advised when generalizing the results of the present study. In addition, as we do not have data on sunlight exposure and use of vitamin D supplements, we were not able to report on the clinical implications of such sources of vitamin D on diabetes. Fourth, we only collected blood samples (ie, serum 25[OH]D_3_ data) at a single time point between the months of August and November, although we did take into account the months during which blood samples were collected in the analysis. Thus, annualized serum 25(OH)D_3_ levels could not be evaluated. Moreover, as we only measured 25(OH)D_3_ levels, we were unable to consider the utility of 25(OH)D_3_ levels in comparison to total 25(OH)D levels with regard to the association between vitamin D and diabetes. Finally, the numbers of both males and females were insufficient to perform analyses stratified by sex; as such, it was necessary to combine males and females to achieve valid comparisons in this study.

In conclusion, results of the present study demonstrate that serum 25(OH)D_3_ levels were inversely associated with diabetes prevalence in a general Japanese population, with a slight inverse association between serum 25(OH)D_3_ levels and HOMA-IR. Our results suggest the possibility that vitamin D_3_ insufficiency impairs glucose homeostasis, likely via insulin resistance, and may cause diabetes. Lifestyle modification that can help to optimize vitamin D_3_ levels in the body may contribute to diabetes prevention. However, larger-scale, longitudinal studies are needed to infer a definite causal relationship.

## SUPPLEMENTARY MATERIAL

eTable 1. Odds ratios for diabetes in participants grouped by sex-specific serum 25-hydroxyvitamin D_3_ quartiles

eTable 2. Odds ratios for diabetes in participants grouped by serum 25-hydroxyvitamin D_3_ levels

eTable 3. Standardized β coefficients between body mass index and ln(HOMA-IR) in the study population
