# Supplementary material for: Serum 25-hydroxyvitamin D3 Levels and Diabetes in a Japanese Population: The DOSANCO Health Study
Source: J Epidemiol. 2023 Jan 5;33(1):31–7. doi: 10.2188/jea.JE20210007 (PMC9727211; doi:10.2188/jea.JE20210007)
Supplement: Supplementary file 1 [file je-33-031-s001.pdf]

eTable 1. Odds ratios for diabetes in participants grouped by sex-specific serum 25-hydroxyvitamin D_3 quartiles

	Serum 25-hydroxyvitamin D_3, ng/mL			
	1st quartile group	2nd quartile group	3rd quartile group	4th quartile group
	Male: ≤18.92 (n=55) (2.08–18.92)	18.93–23.69 (n=55)	23.70–28.91 (n=55)	≥28.92 (n=55) (28.92–58.29)
	Female: ≤17.38 (n=65) (6.55–17.38)	17.39–21.76 (n=65)	21.77–26.28 (n=65)	≥26.29 (n=65) (26.29–78.83)
	(n=120)	(n=120)	(n=120)	(n=120)
Cases of diabetes, n	13	8	9	5
Prevalence, %	10.8	6.7	7.5	4.2
Adjusted odds ratio (95% CI), model 1	3.65 (1.21–11.03)	1.79 (0.55–5.81)	2.04 (0.65–6.42)	1.00 (Reference)
Adjusted odds ratio (95% CI), model 2	4.88 (1.44–16.53)	2.37 (0.67–8.40)	2.47 (0.74–8.25)	1.00 (Reference)
Adjusted odds ratio (95% CI), model 3	4.87 (1.43–16.56)	2.39 (0.67–8.47)	2.50 (0.75–8.39)	1.00 (Reference)

CI, confidence interval; OR, odds ratio.

Diabetes was defined as a fasting plasma glucose level ≥ 126mg/dL and/or a HbA1c level ≥6.5% [reference 24].

Three different logistic regression models were used to calculate odds ratio (95% CI) with the 4th quartile group serving as the reference group: model 1 adjusted for age, sex, and months of blood sample collection; model 2 adjusted for the same covariates used in model 1, in addition to smoking habits, estimated glomerular filtration rate, work status, exercise habits, alcohol intake, protein intake, fat intake, and total dietary fiber intake; model 3 adjusted for the same covariates used in model 2, in addition to body mass index.

eTable 2. Odds ratios for diabetes in participants grouped by serum 25-hydroxyvitamin D_3 levels

	Serum 25-hydroxyvitamin D_3, ng/mL		
	≤19.9 (2.08–19.9) (n=156)	20–29.9 (n=246)	≥30 (30–78.83) (n=78)
Cases of diabetes, n	13	18	4
Prevalence, %	8.3	7.3	5.1
Adjusted odds ratio (95% CI), model 1	2.25 (0.68–7.45)	1.66 (0.53–5.19)	1.00 (Reference)
Adjusted odds ratio (95% CI), model 2	2.97 (0.81–10.85)	2.08 (0.62–7.00)	1.00 (Reference)
Adjusted odds ratio (95% CI), model 3	2.94 (0.80–10.77)	2.09 (0.62–7.02)	1.00 (Reference)

CI, confidence interval.

Diabetes was defined as a fasting plasma glucose level ≥126 mg/dL and/or a HbA1c level ≥6.5% [reference 24].

Three different logistic regression models were used to calculate odds ratio (95% CI) with the 4th quartile group serving as the reference group: model 1 adjusted for age, sex, and months of blood sample collection; model 2 adjusted for the same covariates used in model 1, in addition to smoking habits, estimated glomerular filtration rate, work status, exercise habits, alcohol intake, protein intake, fat intake, and total dietary fiber intake; model 3 adjusted for the same covariates used in model 2, in addition to body mass index

eTable 3. Standardized β coefficients between body mass index and ln(HOMA-IR) in the study population

	Ln(HOMA-IR)	
Overall (N=480)		
Body mass index (kg/m^2)		
Adjusted standardized β coefficient (95% CI)	0.38 (0.33 to 0.44)	p <0.001
Normo-to-prediabetic (N=445)		
Body mass index (kg/m^2)		
Adjusted standardized β coefficient (95% CI)	0.39 (0.33 to 0.44)	p <0.001
Non-obese, normo-to-prediabetic (N=303)		
Body mass index (kg/m^2)		
Adjusted standardized β coefficient (95% CI)	0.39 (0.27 to 0.51)	p <0.001
Obese, normo-to-prediabetic (N=142)		
Body mass index (kg/m^2)		
Adjusted standardized β coefficient (95% CI)	0.33 (0.19 to 0.46)	p <0.001

CI, confidence interval; HOMA-IR, homeostasis model assessment of insulin resistance.

Normo-to-prediabetes was defined as a fasting plasma glucose level <126 mg/dL and a HbA1c level <6.5% [reference 24].

Obesity was defined as a body mass index ≥25 kg/m^2 [reference 25].

A linear regression model was used to examine the standardized β coefficient, adjusting for age, sex, serum 25-hydroxyvitamin D_3, months of blood sample collection, smoking habits, estimated glomerular filtration rate, work status, exercise habits, alcohol intake, protein intake, fat intake carbohydrate intake, and total dietary fiber intake.
